# Solitary Pulmonary Nodule: A Diagnostic Dilemma

**DOI:** 10.1155/2019/5242634

**Published:** 2019-11-21

**Authors:** Pardeep Masuta, Ioana Amzuta

**Affiliations:** ^1^Pulmonary & Critical Care, SUNY Upstate Medical University, Syracuse, USA; ^2^Assistant Professor of Medicine, Pulmonary & Critical Care, SUNY Upstate Medical University, Syracuse, USA

## Abstract

This case describes a woman with a history of tobacco abuse who presented with a dry cough and was found to have an enlarging, 4 cm right upper lobe nodule without lymphadenopathy on CT imaging of the chest. Initial biopsies of the nodule suggested follicular lymphoma, but after obtaining more tissue, the pathology was negative for malignancy and instead showed necrotizing pneumonia. She proceeded to have negative infectious serology and cultures. She had negative rheumatological testing including MPO ANCA, PR-3 ANCA, and rheumatoid factor. She did not have renal failure, hemoptysis, weight loss, arthralgia, or upper airway inflammation. She ultimately underwent a right upper lobectomy, as the nodule was enlarging on repeat imaging. The pathology of the lung showed necrotizing granulomas with giant cells and fibrosis, but lacked active vasculitis or palisading histiocytes, further obscuring the diagnosis. She was conservatively managed with intranasal steroids, cough suppressants and antihistamines given her lack of severe symptoms. The diagnosis was most consistent with granulomatosis with polyangiitis (GPA) presenting as a solitary pulmonary nodule with pathology reflecting a mild degree of disease activity. The patient may manifest further signs of the disease while being monitored clinically.

## 1. Introduction

Granulomatosis with polyangiitis (GPA) is an inflammatory condition that causes a severe, systemic, necrotizing vasculitis. The most common manifestations include constitutional symptoms such as fevers, malaise, and weight loss; respiratory tract symptoms such as hemoptysis, epistaxis and pulmonary nodules; and progressive renal failure with hematuria. The initial presentation varies significantly between patients, and this can make the diagnosis particularly difficult as the symptoms can resemble other diseases such as eosinophilic granulomatosis with polyangiitis (EGPA), microscopic polyangiitis (MPA), infections, and malignancies.

## 2. Case Presentation

A 66-year-old female with a distant 15 pack year smoking history presented to the pulmonary clinic for evaluation of a 4 cm right upper lobe mass found on a CT thorax. She had a dry cough with occasional fatigue and night sweats that began about a year earlier. She denied recent travel, weight loss, hemoptysis, epistaxis, wheezing, joint pain, hematuria, peripheral edema, orthopnea, easy bruising or alopecia. Her vitals and physical exam were within normal limits on room air.

**Figure 1:**
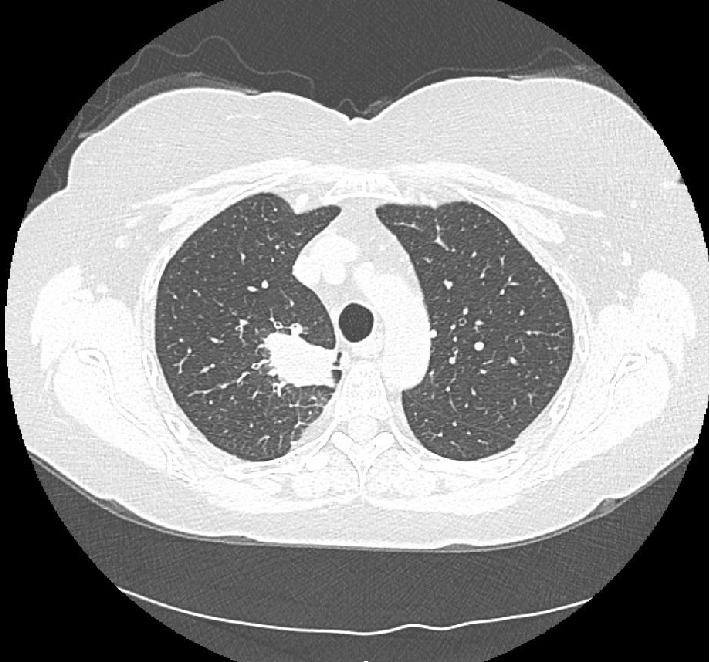
CT scan of the thorax showing a large right upper lobe spiculated nodule.

**Figure 2:**
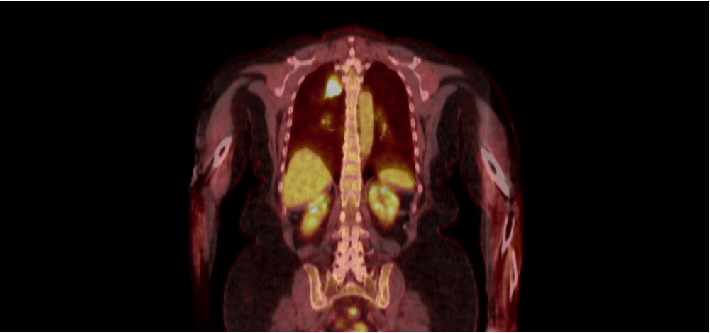
PET CT showing a hypermetabolic right upper lobe nodule.

She had been treated as an outpatient for presumed community acquired pneumonia when her symptoms first began. Follow up chest X-ray showed a persistent opacity in the right upper lobe and a CT scan showed a large, spiculated 4 cm right upper lobe mass (Figure 1). It was hypermetabolic with an SUV of 19 on PET CT (Figure 2). Her pulmonary function tests revealed a moderate reduction in diffusion capacity without obstruction or restriction. Her FEV1/FVC ratio was 71%. FVC was 90% of predicted at 2.94 L. FEV1 was 83% of predicted at 2.08 L. Her TLC was 94% of predicted at 4.77 L and vital capacity was 90% of predicted at 2.94 L. Her DLCO was 54% of predicted at 12.8.

She underwent a bronchoscopy and EBUS guided needle aspiration of the mass. Pathology revealed a population of CD10 positive lymphocytes suggestive of follicular lymphoma.

**Figure 3:**
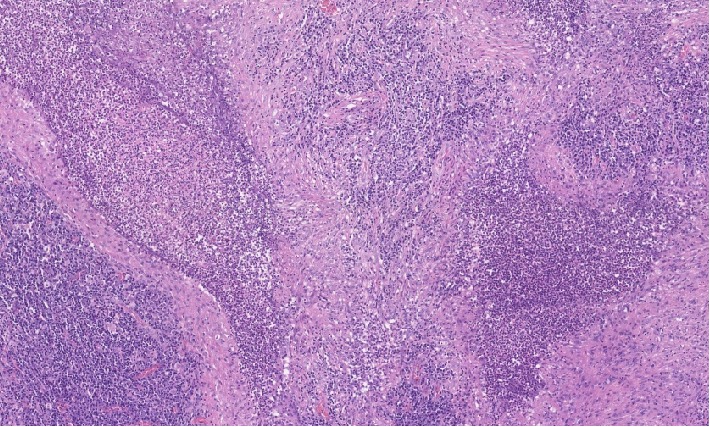
High power histopathology of lung showing necrotizing inflammation.

**Figure 4:**
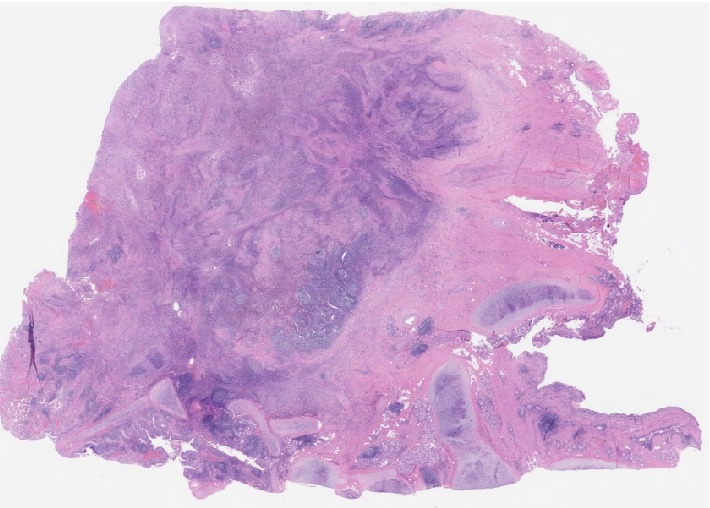
Low power histopathology of lung showing necrotizing inflammation.

A CT guided biopsy was performed to obtain additional tissue for histological and genetic testing. It surprisingly turned out to be negative for lymphoma but instead showed necrotizing pneumonia. There were areas of necrosis with surrounding fibrosis. Neoplastic cells were not identified (Figures 3 and 4). AFB cultures and fungal cultures were negative. Quantiferon, aspergillus galactomannan, histoplasma, blastomyces and coccidioides antibodies were also negative. ANCA serology including MPO (<9 u/mL) and PR3 (<3.5 u/mL) testing was negative. Rheumatoid factor, C3 and C4 were also negative. Her ANA speckled pattern was 250. Her urinalysis had 3+ protein with a normal protein/creatinine ratio. There were no casts. Her BMP and CBC were within normal limits without eosinophilia.

The patient's cough persisted despite conservative treatment with intranasal steroids, cough suppression, and antihistamines. ENT evaluation with nasopharyngolaryngoscopy (NPL) did not reveal upper airway inflammation or ulceration. A repeat CT scan of the thorax showed that the mass was enlarging.

A video assisted thoracoscopy (VAT) with right upper lobectomy was performed. It revealed a right upper lung mass with multiple adhesions to the pleura. The pathology revealed necrotizing granulomas with eosinophils and giant cells. There were no malignant cells, active vasculitis, or organisms found.

The favored diagnosis was granulomatosis with polyangiitis (GPA).

## 3. Discussion

Granulomatosis with polyangiitis is often associated with a positive cytoplasmic ANCA and manifests with constitutional symptoms, upper/lower respiratory tract symptoms, and renal symptoms [1–5].

Diagnosis can be difficult as the spectrum of disease varies greatly between people. Without treatment, the disease can cause multi-organ destruction. Patients can have fever, myalgia, and weight loss for months without signs of organ involvement [1]. Upper airway symptoms such as epistaxis, recurrent sinusitis, or saddle-nose deformity can be helpful to differentiate it from other forms of vasculitis such as microscopic polyangiitis (MPA) [1–3]. Up to 90 percent of patients with GPA have some form of upper respiratory involvement compared with 35 percent of those with MPA [4]. 25 percent of patients can have pulmonary involvement which can cause cough, stridor, wheezing, or hemoptysis from alveolar hemorrhage. Renal involvement is common in GPA, presenting as hematuria and progressive renal failure. Active glomerulonephritis is usually demonstrated in up to 85 percent of patients within the first two years of disease onset [5]. Diagnosis is achieved by demonstrating this constellation of symptoms along with imaging, immunological, and histological findings.

ANCA testing should be performed on any adult patient presenting with symptoms suspicious for vasculitis, but the absence of ANCA does not exclude the diagnosis of GPA. Approximately 90 percent of patients with GPA or MPA are ANCA positive [6]. PR3-ANCA is positive in 80–90 percent of GPA patients with the remainder being MPO-ANCA. The disease activity can correlate with the degree of positivity of PR3-ANCA at the time of sampling. Less than 10% of patients with active, generalized GPA will have negative ANCA titers. In respiratory limited subsets, up to 40 percent of patients may be ANCA negative while up to 80 percent of patients with renal-limited vasculitis are actually positive for MPO-ANCA [6–8].

A hypermetabolic, solitary, and spiculated nodule can be malignant (e.g., adenocarcinoma, small cell carcinoma or metastatic), infectious (e.g., tuberculosis, histoplasmosis or cryptococcosis), or inflammatory in nature. Common causes of inflammatory nodules include sarcoidosis, rheumatoid nodules and vasculitides such as GPA.

Sarcoidosis is a common inflammatory condition that usually affects young and middle aged patients between 20 and 40. It can involve multiple organ systems, but affects the lungs in 90% of cases. It most commonly presents with mediastinal lymphadenopathy and bilateral reticular opacities on chest X-ray imaging. Nodular sarcoid is a rare pulmonary manifestation of sarcoidosis. Sarcoid nodules usually present as bilateral, multiple opacities occupying the perihilar or peripheral lung zones on a chest CT. These lesions may be accompanied by mediastinal lymphadenopathy and galaxy sign (small satellite nodules that border the periphery of larger nodules) [3]. This patient did not have significant lymphadenopathy, systemic symptoms, or other pulmonary nodules.

Rheumatoid nodules are one of the most common pulmonary manifestations of rheumatoid arthritis [9]. They usually do not cause pulmonary symptoms despite being about 1–3 cm in size. They can cavitate on very rare occasions. During that time, they may be prone to becoming infected, increasing the risk for pneumonia and lung abscesses. Other common pulmonary manifestations of rheumatoid arthritis include interstitial lung disease and pleural disease. These patients can have other symptoms of rheumatoid arthritis such as peripheral polyarthritis and joint destruction. Treatment includes controlling the underlying rheumatoid arthritis with NSAIDS and immunomodulatory therapy [9, 10].

Although the most common imaging abnormality in patients with GPA is multiple, bilateral pulmonary nodules, there is significant variability [6, 11–13]. Findings include bronchial wall thickening, effusions, consolidations, and ground glass opacities [11–13]. These can be localized or diffused; with or without adenopathy. Nodules can vary anywhere from 3 mm to over 30 mm and from solid to cavitating. These are often hypermetabolic on 18F-fluorodeoxyglucose PET/CT, making it difficult to differentiate between malignancy, pneumonia or noninfectious inflammatory conditions [6, 12, 13].

The diagnosis of GPA should be confirmed by a biopsy of the suspected site of active disease whenever possible. The most commonly affected sites are the kidneys followed by the lungs. The usual findings in either site are necrotizing granulomatosis, seen as foci of necrosis and granulomas with palisading histiocytes (with the long axis perpendicular to the necrotic center) and vasculitis [7, 11, 14, 15]. Renal biopsy findings usually parallel the severity of the patient's disease on clinical presentation [7, 8]. Findings can show mild focal and segmental glomerulonephritis in patients with asymptomatic hematuria to diffuse, crescentic glomerulonephritis and necrosis in patients with renal failure. Arteritis and granulomas are occasionally seen [7, 8]. This patient had very mild symptoms and did not have any signs of renal failure; therefore, a renal biopsy was not performed. The pulmonary lesions in GPA are usually firm, hemorrhagic, yellowish-gray nodules with an average size of 2.4 cm on gross inspection [8, 15]. There may be central necrosis which is noncaseating and friable. Transmural infiltration of blood vessels with inflammatory cells can be noted on microscopy. In patients presenting with alveolar hemorrhage, pulmonary capillaritis is a key finding [15]. The most abundant infiltrating inflammatory cells include neutrophils, lymphocytes, and multinucleated giant cells. There may be occasional eosinophil infiltration seen, but it is usually without peripheral eosinophilia. Small areas of organizing pneumonia with microabscesses could also be noted [15]. Negative stains and cultures are needed to rule out infectious etiologies such as tuberculosis or histoplasmosis.

The lack of classical symptoms, negative ANCA serology, and no active vasculitis on histology complicated the diagnosis in this patient. This likely represented a mild degree of disease activity; evidenced by her relative lack of extrapulmonary symptoms [11]. She may develop further signs of the disease going forward. This patient demonstrates a rare case of GPA manifesting as a solitary pulmonary nodule and the importance of maintaining a broad differential based on clinical and imaging findings for this potentially life threatening disease.
